# Management of Soft Tissue Deficiency Around an Anterior Implant Placed During the Adolescent Period: A Case Report of 30 Months Follow-Up

**DOI:** 10.7759/cureus.107429

**Published:** 2026-04-20

**Authors:** Duygu Yaman, Burak Saygın, Beyza Bozoklu, Nülüfer Demir, Sina Saygılı, Tonguç Sülün

**Affiliations:** 1 Periodontology, Istanbul University Faculty of Dentistry, Istanbul, TUR; 2 Prosthodontics, Istanbul University Faculty of Dentistry, Istanbul, TUR

**Keywords:** adolescence, anodization, anterior implant, customized abutment, digital workflow, esthetic rehabilitation, peri-implant soft tissue augmentation

## Abstract

This clinical report describes the interdisciplinary rehabilitation of a 21-year-old male patient who presented with esthetic concerns due to an anterior implant placed during adolescence following traumatic tooth loss. Clinical and radiographic examination revealed the presence of an implant in infraposition at tooth #21, along with vertical and horizontal peri-implant soft tissue deficiencies. The patient underwent two sequential soft tissue augmentation procedures using de-epithelialized gingival grafts to improve the volume and stability of the mucosa. A customized abutment was digitally designed and fabricated, considering the buccal malposition of the implant, and provisionalized to allow soft tissue conditioning. Anodization techniques were employed to minimize the grayish shine-through effect of the titanium abutment. Final restoration was completed using a monolithic zirconia crown, shade-matched using Matisse.AI (LabMatisse, Veenendaal, the Netherlands). This case underscores the challenges associated with implant placement during adolescence and highlights the importance of soft tissue management, abutment customization, and the integration of digital prosthetic workflows to achieve mid-term esthetic and functional success.

## Introduction

Adolescence is the phase of life that begins with the start of puberty and generally ends around age 19 [1]. Permanent tooth loss due to trauma is a common cause of tooth loss during this period [2,3]. It may cause problems such as alterations in speech, migration of adjacent teeth into the gap, esthetic concerns, and loss of self-confidence. Treatment options include removable or full dentures and fixed dental restorations [4]. Although removable dentures are a commonly preferred treatment option, in contemporary dentistry, teeth or implant-supported fixed restorations and long-term solutions are particularly preferred for addressing problems in the esthetic regions of young patients.

Dental implants are considered to be a predictable replacement of missing teeth [5]. The success of implant treatment requires proper selection of the patient and site [6]. To date, there is no consensus regarding implant placement during adolescence, particularly with respect to optimal timing thresholds relative to skeletal maturity, the most reliable method for assessing growth completion (e.g., hand-wrist radiographs, cephalometric analysis, cervical vertebral maturation), and the magnitude and predictability of infraposition risk in different regions of the jaws. Replacing a missing tooth with an implant in a growing patient is believed to help preserve bone volume, with good vascularization and strong bone healing potential during this period being important contributing factors [7,8]. Nevertheless, this potential advantage must be weighed against the well-documented risks of infraposition, esthetic compromise, and occlusal discrepancies that may develop as the surrounding dentoalveolar structures continue to grow [9,10].

During adolescence, the jaws are still actively undergoing changes. The maxilla is particularly more affected by growth in the vertical direction compared to the mandible, and this continues until early adulthood (approximately 17-20 years of age in females and 18-22 years in males, though residual vertical changes may persist into the third decade of life) [9,11]. Therefore, assessment of skeletal maturity through methods such as hand-wrist radiographs or cervical vertebral maturation analysis is recommended prior to implant placement in the anterior maxilla, rather than relying solely on chronological age. Furthermore, it has been suggested that the anterior maxilla may be the most challenging area for early implant placement due to the unpredictable nature of growth in this region [10]. Since periodontal tissues such as the periodontal ligament and cementum do not form around the implant, there is no microenvironment to facilitate its movement within the jaw. Instead, the implant and surrounding bone establish an ankylosis-like rigid connection, rendering the implant unable to keep pace with the growth of the surrounding tissues. Consequently, as the adjacent dentoalveolar structures continue to develop vertically while the implant remains stationary, progressive infraposition develops, resulting in a discrepancy in the gingival margin levels between the implant site and the neighboring teeth [9,12]. Some clinical studies have reported that implants remain in an infraocclusal position ranging from 0.1 to 4.5 mm [9,13,14]. The magnitude of infraposition is influenced by factors such as the patient's age at implant placement, residual growth potential, craniofacial growth pattern (e.g., hyperdivergent facial type), implant site, and the presence or absence of anterior tooth contacts. This may result in esthetic concerns, particularly in the maxillary anterior region. However, there is no consensus on the management of this clinical scenario.

The presence of adequate hard and soft tissue is essential for maintaining peri-implant health [15,16]. Regarding the relationship between these tissues, sufficient horizontal soft tissue thickness, reported to be at least 2 mm, has been shown to be associated with reduced marginal bone loss, although this relationship may be influenced by confounding factors such as implant platform design and prosthetic configuration [17]. In fact, long-term clinical studies have reported that healthy peri-implant mucosal tissues can remain stable even in areas where the buccal bone is absent [18,19]. Therefore, it has recently been argued that preserving soft tissue health and intervening when necessary are key elements in modern implantology [20].

The aim of this case report is to describe the interdisciplinary rehabilitation of a young patient with an anterior implant placed during adolescence, which subsequently remained in relative infraposition. The rehabilitation involved two sequential soft tissue augmentation procedures, a digitally designed customized titanium abutment incorporating anodization to address esthetic concerns, and a final monolithic zirconia restoration. Outcomes are reported over a 30-month follow-up period.

## Case presentation

A 21-year-old systemically healthy male patient (American Society of Anesthesiologists (ASA) I, no medications, no smoking) presented to our clinic with esthetic concerns regarding his anterior teeth in February 2024. At the time of the visit, the patient had a temporary removable prosthesis on tooth #21 and expected to improve his smile with a fixed esthetic restoration. The anamnesis revealed that he lost the affected tooth due to trauma at the age of 15, and one month after the loss, an endosseous implant (Osstem TSIII SA, 4.5×11.5 mm, Osstem Implant Co., Seoul, South Korea) was placed without prior skeletal maturity assessment. No grafting or orthodontic treatment was performed before or during implant placement. The implant-supported crown was first delivered shortly after implant placement; it was fully remade in August 2023 due to progressive esthetic concerns related to infraposition and subsequently became decemented and was lost in the following months.

**Figure 1:**
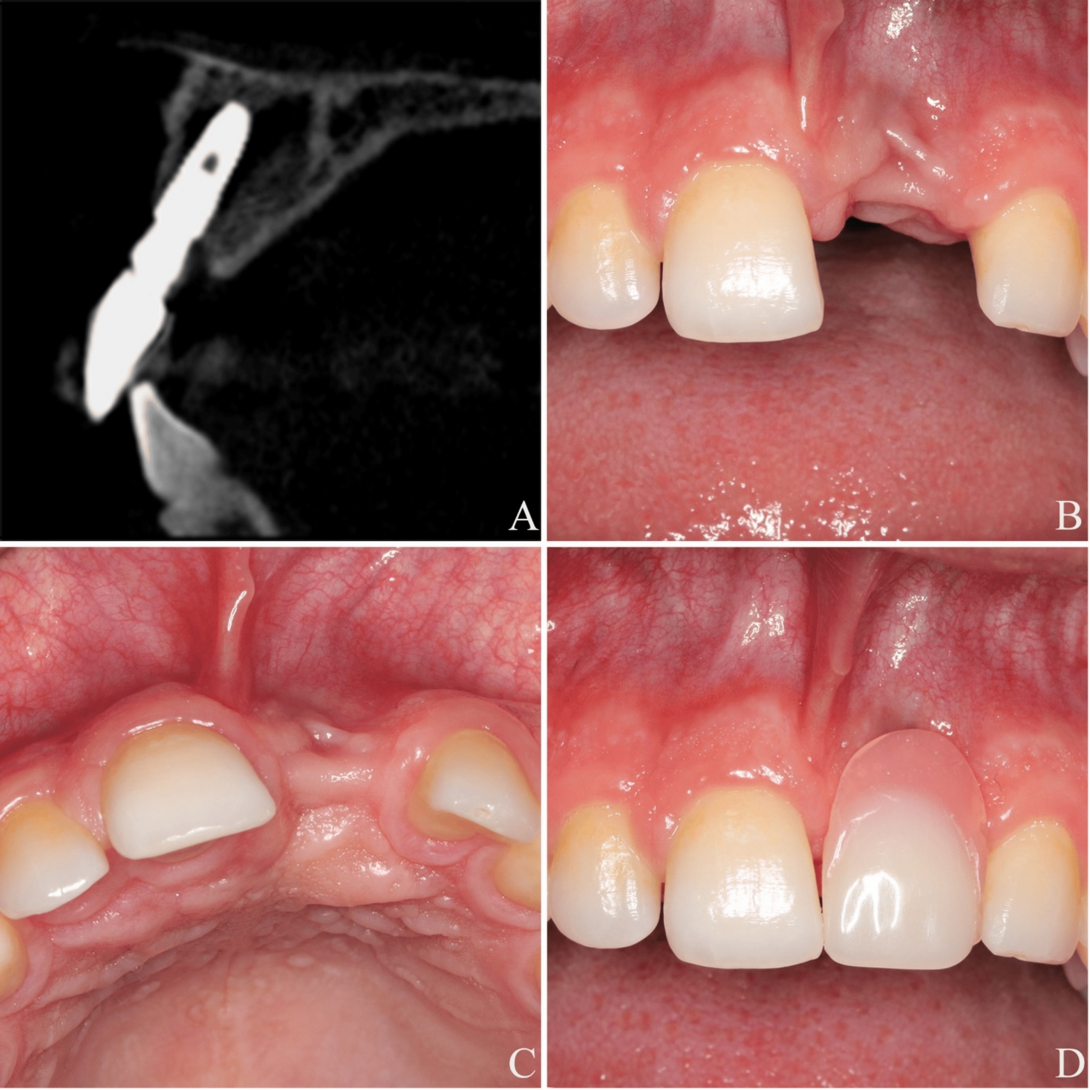
(A) Sagittal CBCT view of anterior implant #21, December 2023. (B) Frontal view at baseline. (C) Occlusal view at baseline. (D) Frontal view at baseline with removable prosthesis CBCT: cone beam computed tomography

Clinical and radiological examinations revealed the presence of infraposition of an implant body in the area of tooth #21. Periapical radiographic measurements revealed that the implant platform was located approximately 6 mm apical to the cemento-enamel junction (CEJ) of tooth #11 and 7 mm apical to the CEJ of tooth #22, indicating an approximately 5-6 mm positional discrepancy compared to the recommended 1 mm apical position described by Buser et al. [21]. The cone beam computed tomography (CBCT) scan, taken in December 2023 with the implant-supported crown that had been renewed in August 2023 due to esthetic concerns which had been lost in the following months, showed a thin buccal bone plate on the coronal part of the implant, resulting from too buccal placement (Figure 1A). Additionally, vertical and horizontal deficiencies in the peri-implant soft tissue were identified (Figure 1B-1C). According to the classification proposed by Zucchelli et al., the defect was categorized as a class IV facial peri-implant soft tissue deficiency, given the buccal malposition of the implant head relative to the profile of the adjacent teeth [22]. Baseline peri-implant assessment revealed probing depths (PD) of 8 mm at the disto-buccal and disto-palatal aspects of the implant, with bleeding on probing (BOP) positive at the distal sites. The remaining sites showed no BOP and PD above 3 mm.

**Figure 2:**
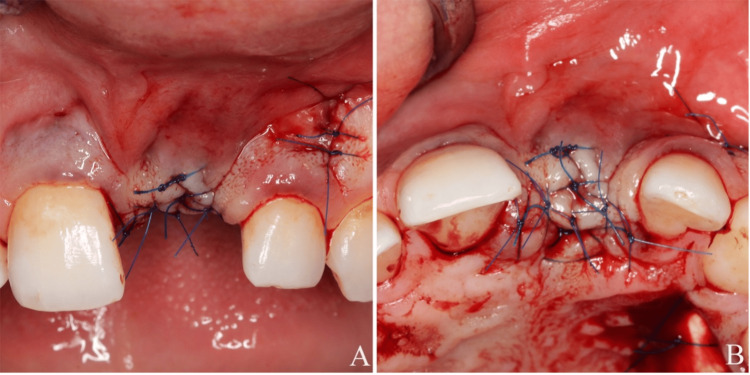
(A) Frontal view after the first operation. (B) Occlusal view after the first operation

Following non-surgical periodontal and peri-implant therapies (supragingival scaling of all tooth surfaces and non-surgical peri-implant debridement were carried out, along with oral hygiene instructions) and discussion of the treatment options with the patient, a connective tissue graft procedure was planned to increase the soft tissue volume on the buccal side. In February 2024, a partial-thickness flap was elevated in the buccal area of the implant site through an incision placed slightly palatal to the alveolar crest. This approach was selected to maintain the integrity of the overlying soft tissue flap, thereby enhancing graft vascularization through a dual blood supply from both the periosteal bed and the overlying flap while minimizing surgical morbidity by avoiding vertical releasing incisions and preserving the existing soft tissue architecture. A de-epithelialized gingival graft (de-ep GG) was then positioned and secured with the sutures (6/0 polyvinylidene fluoride, Trofilene, Dogsan, Turkiye). The flap was then repositioned over the graft and left to heal by primary intention (Figure 2A-2B).

**Figure 3:**
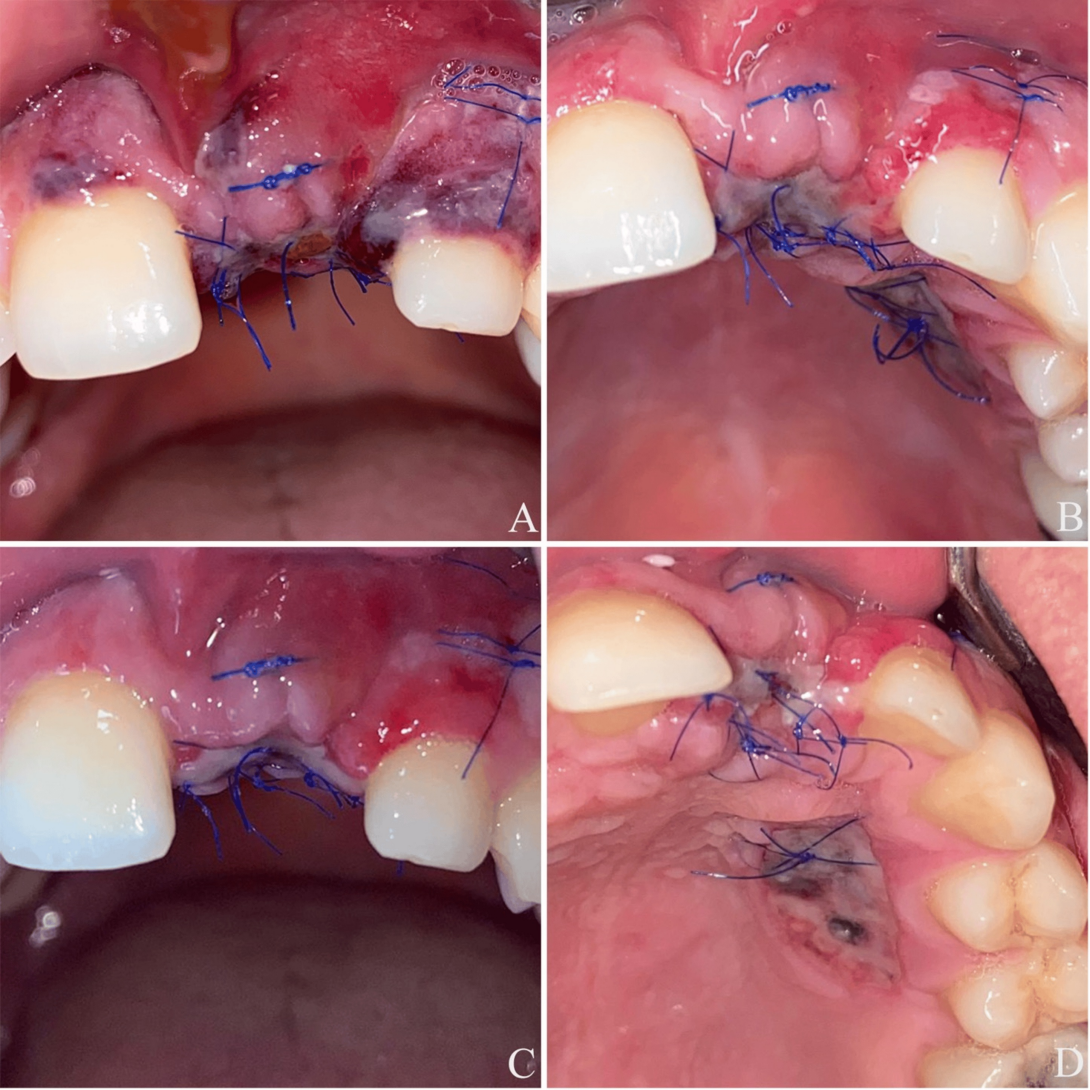
(A) Two days after the first operation. (B) Five days after the first operation. (C) Seven days after the first operation. (D) Seven days after the first operation

During the healing process, the patient was monitored on the second, fifth, and seventh days, and both recipient and donor areas exhibited secondary healing during the first week (Figure 3A-3D). The patient was prescribed clindamycin 600 mg (IM) 2×1 for three days, and the recipient-donor areas were irrigated with chlorhexidine (0.12%) every other day between postoperative days 5 and 10. After two weeks, sutures were removed, and both surgical sites healed and were absent of inflammation (e.g., suppuration, bleeding) with an immature but clinically healthy peri-implant soft tissue.

**Figure 4:**
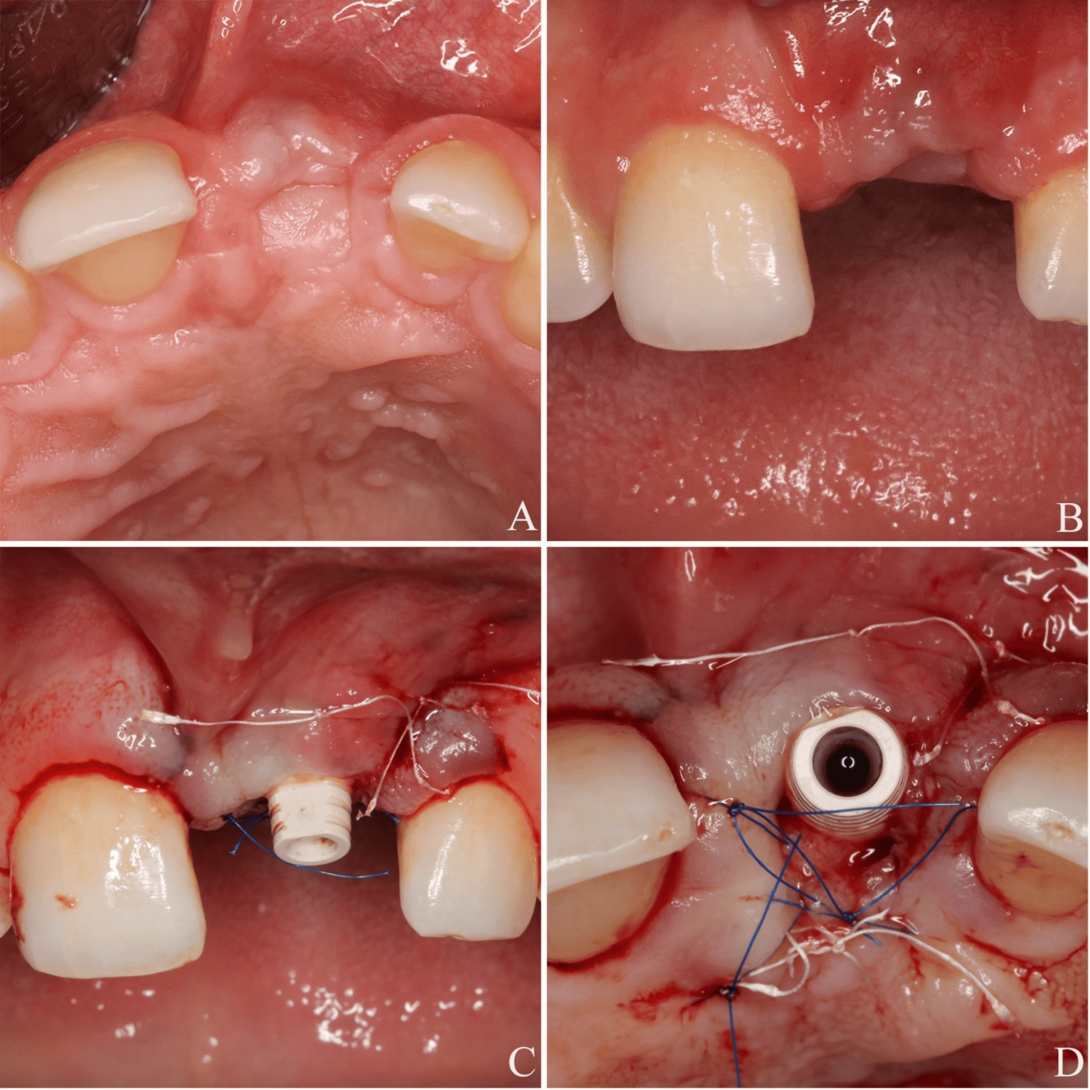
(A) Occlusal view before the second operation, showing disto-buccal concavity. (B) Frontal view before the second operation. (C) Frontal view after the second operation. (D) Occlusal view after the second operation

Two months after the first operation, following the maturation of the operated area, the soft tissue was reassessed. Clinical examination revealed a keratinized mucosa width of less than 2 mm on the buccal aspect of the implant site, with a persistent disto-buccal concavity (Figure 4A-4B). An additional soft tissue augmentation procedure with de-ep GG was therefore planned to address both findings at the time of abutment connection. The implant was surgically uncovered, and a polyetheretherketone (PEEK) temporary abutment was placed, which revealed that the implant had been placed too buccally (Figure 4C-4D). A free gingival graft was harvested from the palate, de-epithelialized, and placed between the buccal soft tissue and the temporary abutment. This additional tissue was intended to reinforce the buccal soft tissue volume and resist potential mucosal recession resulting from the buccal malposition of the implant. The graft was fixed to the overlying soft tissue with sutures (6/0 polyvinylidene fluoride, Trofilene), and the tissues were left to heal transgingivally.

**Figure 5:**
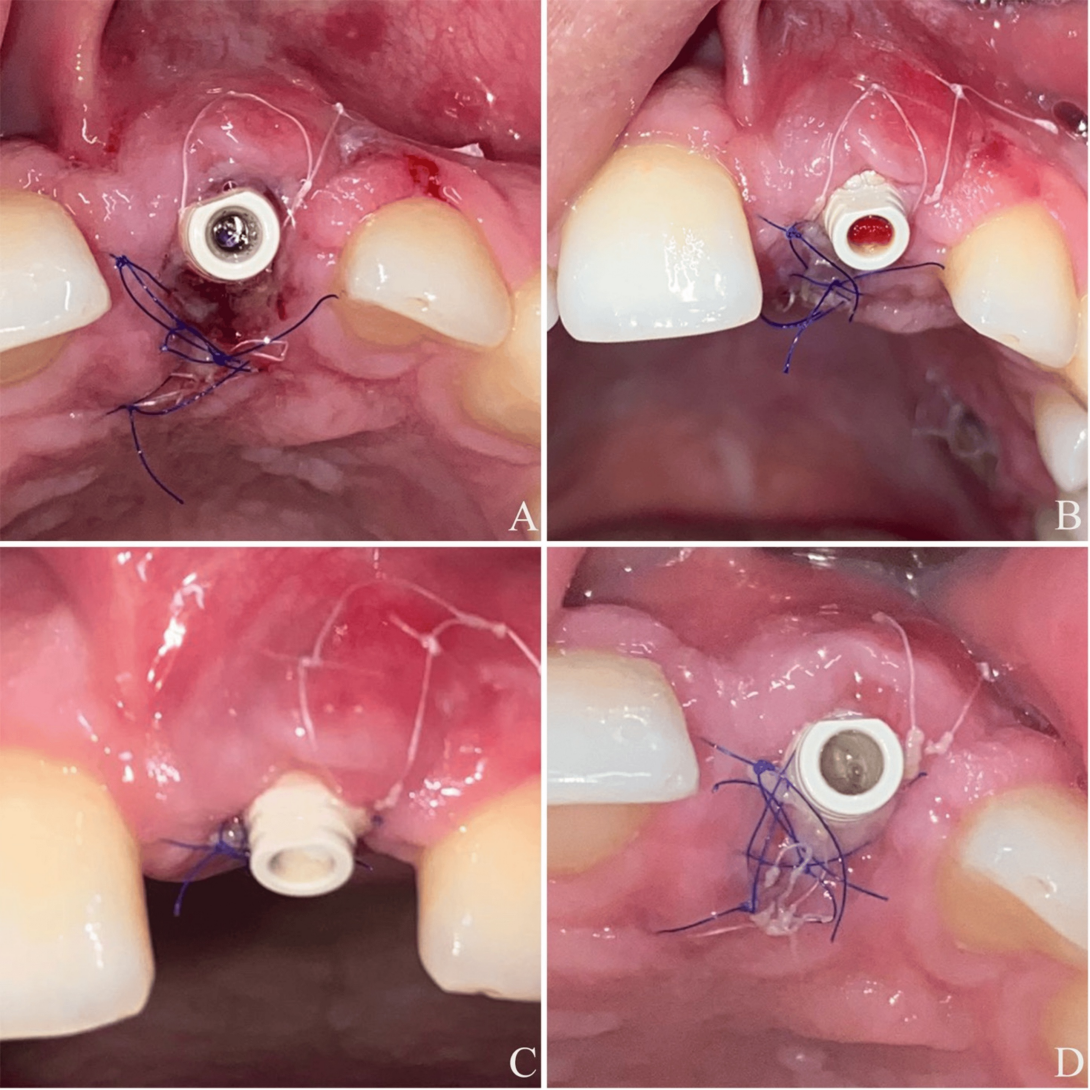
(A) Two days after the second operation. (B) Five days after the second operation. (C) Ten days after the second operation. (D) Ten days after the second operation

The healing was uneventful after the second operation, with clinically satisfactory soft tissue maturation confirmed by the absence of inflammation, no BOP, stable mucosal margins, and adequate graft integration (Figure 5A-5D). Based on these objective criteria, the patient was scheduled for the prosthetic phase of the therapy one month after the second operation.

**Figure 6:**
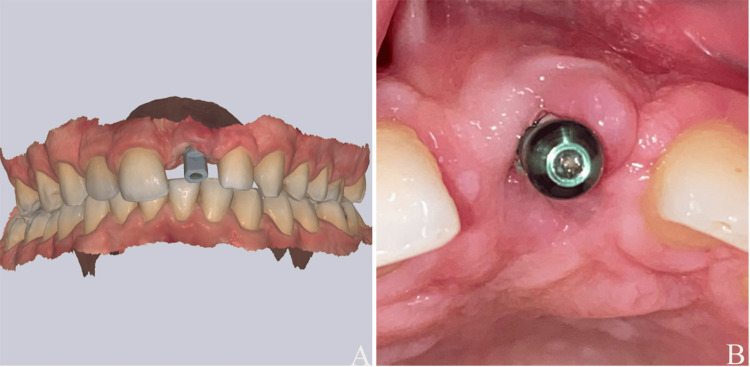
(A) Digital impression. (B) Open-tray impression coping used a substitute for the gingiva former

To initiate prosthetic soft tissue management and the final restoration, a comprehensive digital impression protocol was executed using an intra-oral scanner (Alliedstar AS 200E, Shanghai, China) (Figure 6A). Since the manufacturer's maximum available gingiva former height is 7 mm, it was determined that even this component would be insufficient for the 6-7 mm apical depth of the implant platform. Consequently, an 11 mm open-tray impression coping (Osstem TS System, Regular, Seoul, South Korea) was utilized as a substitute for a gingiva former to maintain and condition the soft tissue (Figure 6B). During the digital workflow, the open-tray coping was first removed to capture the soft tissue and emergence profile. Although a standard digital scan body (TS Scan Body, Regular, Short-7mm, Osstem, Seoul, South Korea) was initially considered, its 7 mm height resulted in it being submerged below the level of the peri-implant mucosa. Therefore, a longer version of the scan body (TS Scan Body, Regular, Long-12mm, Osstem, Seoul, South Korea) was employed to ensure adequate visibility above the mucosal margin and maintain technical transparency regarding the implant platform position (Figure 6A). The protocol was finalized with a digital bite registration. It is important to note that the open-tray coping served strictly as a tissue management aid; no conventional impressions were taken at this stage.

**Figure 7:**
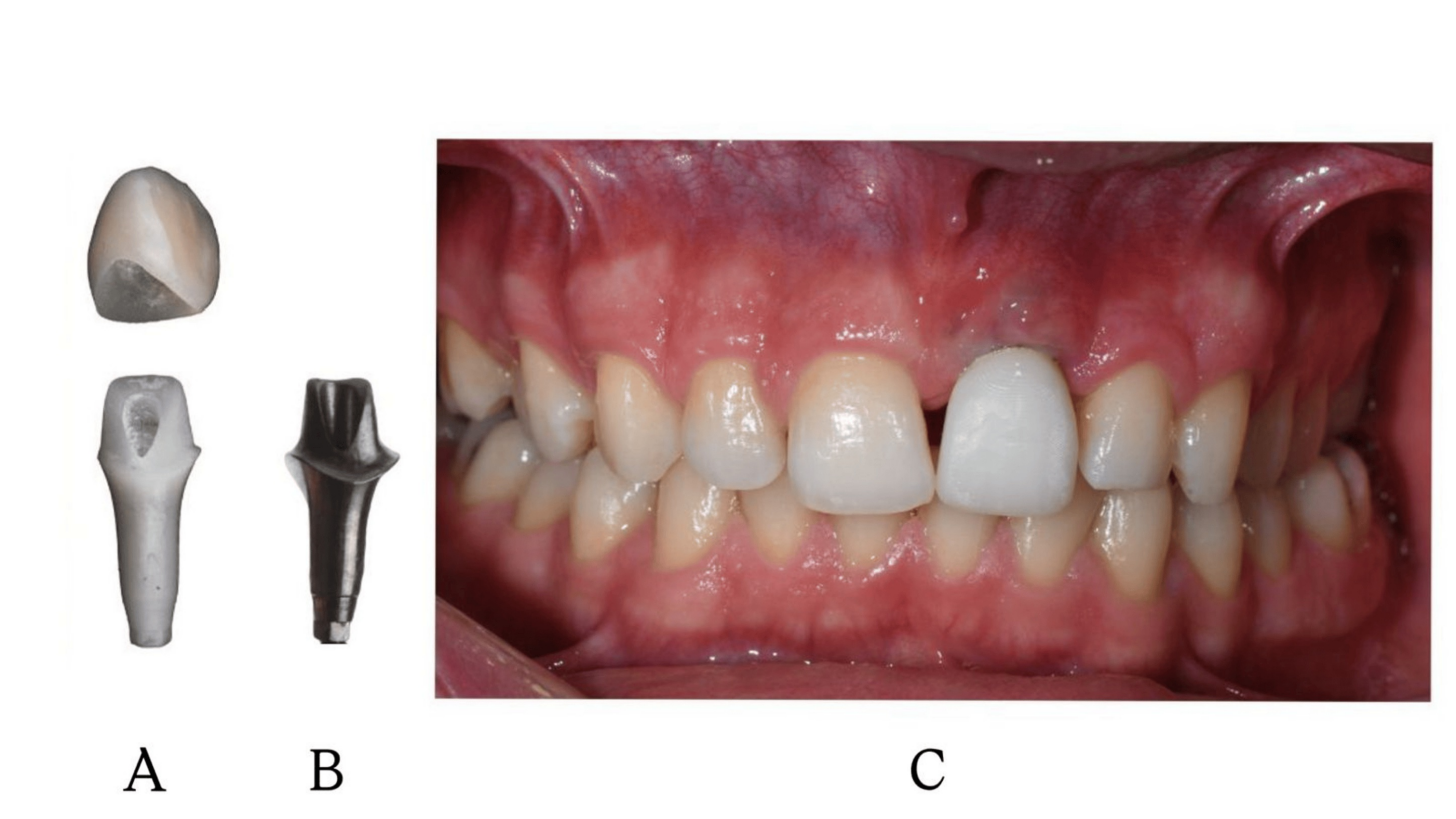
(A) Temporary customized abutment. (B) Final customized abutment fabricated by milling. (C) The permanent customized abutment was screwed into place and a temporary crown fabricated from PMMA was cemented with a non-eugenol temporary cement PMMA: polymethyl methacrylate

During the design phase, the files were imported into Exocad (DentalCAD 3.2, Darmstadt, Germany). Since the maximum available height for the manufacturer's standard Ti-base abutments is only 4 mm, which was insufficient to compensate for the clinical depth in this case, it was decided to fabricate a customized abutment. Following the design of the customized abutment and the final crown, a prototype was first produced using a 3D printer (Max UV, Asiga, Sydney, Australia) and evaluated through an intra-oral try-in (Figure 7A).

During the intra-oral try-in, the fit of the abutment and crown was evaluated. To harmonize the morphology with tooth #11, the incisal edge of the crown was adjusted using a disc. These intra-oral adjustments were subsequently transferred to the design software, and the final customized abutment was fabricated using the milling method (Figure 7B). In accordance with the "Zero Bone Loss" concepts proposed by Linkevicius, the subcritical contour, extending from the implant platform to 2 mm below the gingival margin, was designed to match the narrow, non-compressing shape of the open-tray impression coping [23]. This approach aimed to preserve the surrounding tissues and prevent potential graft instability caused by excessive compression on the recently augmented area. Conversely, the final 2 mm of the abutment, representing the critical contour, was designed with a convex profile. This strategic modification was implemented to shape the gingival zenith and mimic the cervical morphology of the adjacent tooth (#11), thereby optimizing the final esthetic emergence profile without compromising the underlying biological stability.

The customized abutment was seated and secured at 30 Ncm. To ensure biological safety during the provisional phase, Teflon tape was used to prevent excess cement extrusion into the peri-implant sulcus. A temporary crown fabricated from polymethyl methacrylate (PMMA) was then cemented with a non-eugenol temporary cement (Cavex, Haarlem, the Netherlands). Excess cement was meticulously removed through a two-stage cleaning procedure using a sharp curette (Figure 7C).

**Figure 8:**
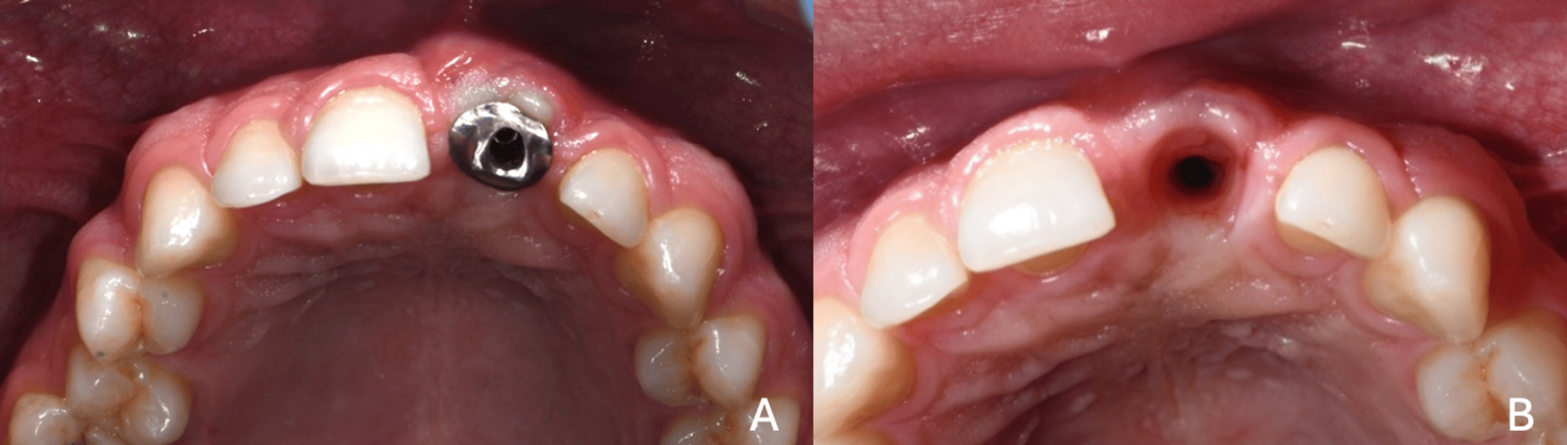
(A) Final customized abutment. (B) Tissue maturation after two months with the final abutment

The patient was monitored for approximately two months to allow for soft tissue maturation and shaping. During this period, clinical parameters were objectively evaluated through standardized clinical photography and periodontal measurements (Figure 8A-8B). At the two-month evaluation, PD of 5 mm were recorded at the disto-buccal and disto-palatal aspects with no BOP, and the remaining sites maintained PD of 3 mm without bleeding, confirming adequate soft tissue stability prior to proceeding with the final restoration.

Following the two-month maturation period, the permanent restoration phase was initiated. Although a digital workflow had been initially employed for the design of the customized abutment, a hybrid approach was adopted for the final crown to ensure subgingival accuracy. The temporary crown was removed, and a conventional impression was obtained over the abutment using a polyether material. This decision was made due to the potential limitations of intra-oral scanners in capturing deep subgingival contours and to avoid applying unnecessary stress to the already torqued abutment. To ensure clear margin visibility, retraction was performed prior to the impression using a retraction cord (#00, Ultradent, South Jordan, Utah, United States).

**Figure 9:**
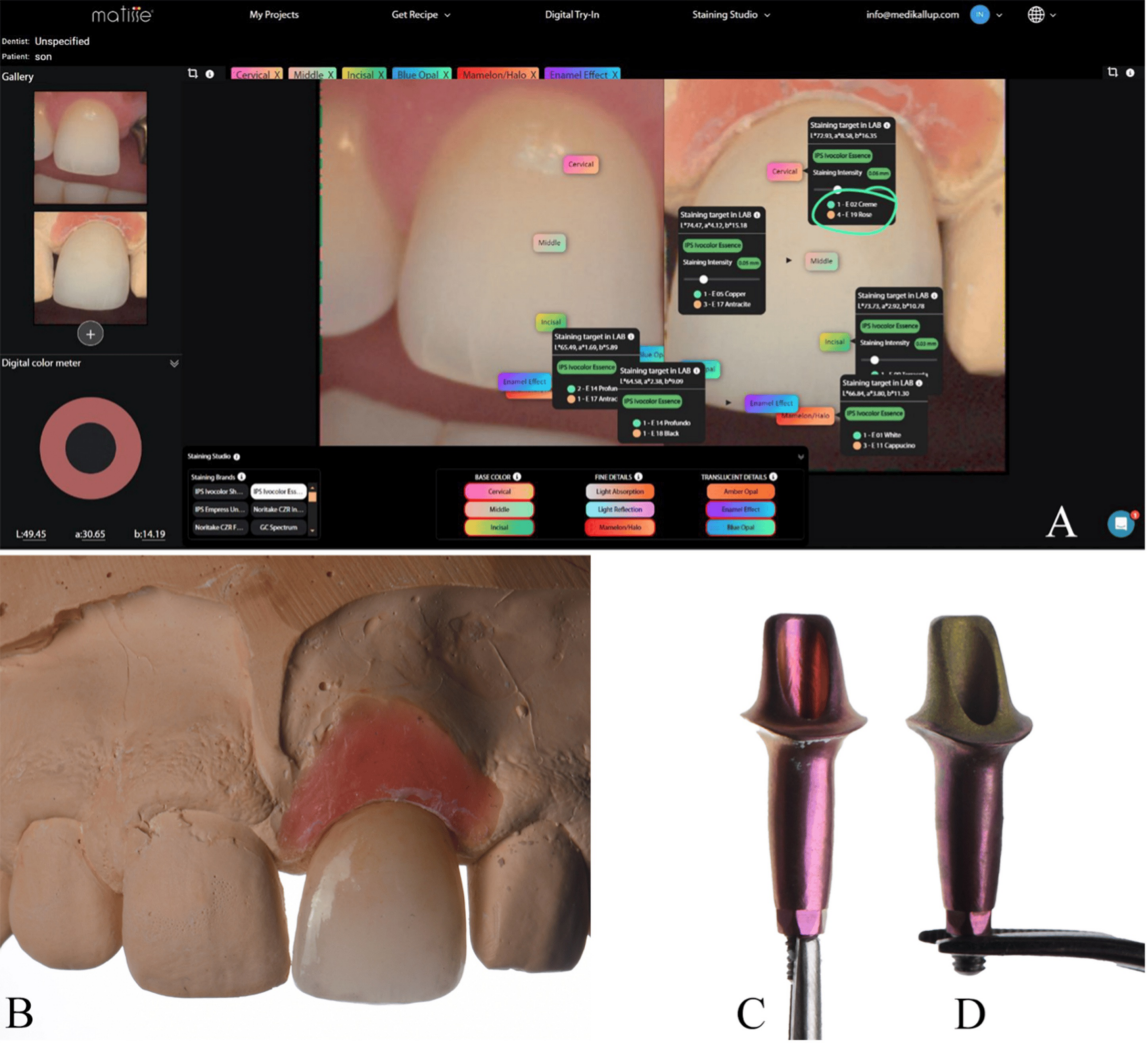
(A) Porcelain recipe (Matisse.AI (LabMatisse, Veenendaal, the Netherlands)). (B) After performing a facial cut-back, porcelain was applied to the facial surface, according to the recipe provided by the software. (C) The abutment was anodized in sulfuric acid solution with an 80V current, giving it a pink color. (D) A 50V current was applied, resulting in a yellow color

To complete the hybrid workflow, the final shade was determined using the OptiShade (Smile Line SA, St-Imier, Switzerland) digital shade-matching device and polarized photography. The digital shade-matching workflow involved uploading the data into Matisse.AI (LabMatisse, Veenendaal, the Netherlands) to generate a precise porcelain mixing recipe (Figure 9A). The restoration was initially milled from a monolithic zirconia block, followed by a facial cut-back and porcelain layering according to the software-generated recipe (Figure 9B).

During the clinical try-in, a grayish reflection from the abutment was visually observed within the thin-phenotype soft tissue lacking bone support. To address this, the abutment was removed for the anodization process. The surface was decontaminated in an ultrasonic bath, immersed in a sulfuric acid solution, and an 80V current was applied for 10 seconds to achieve a pink anodic coating (Figure 9C). Upon re-trying, a pink reflection was noticed beneath the crown. To resolve this, the coronal portion of the abutment was modified with a yellow hue. The abutment was removed once more, and during this interval, the initial 3D-printed prototype was re-inserted to maintain the soft tissue architecture. After decontamination, the coronal surface was sandblasted and immersed in the solution with a 50V current applied for 10 seconds (Figure 9D).

**Figure 10:**
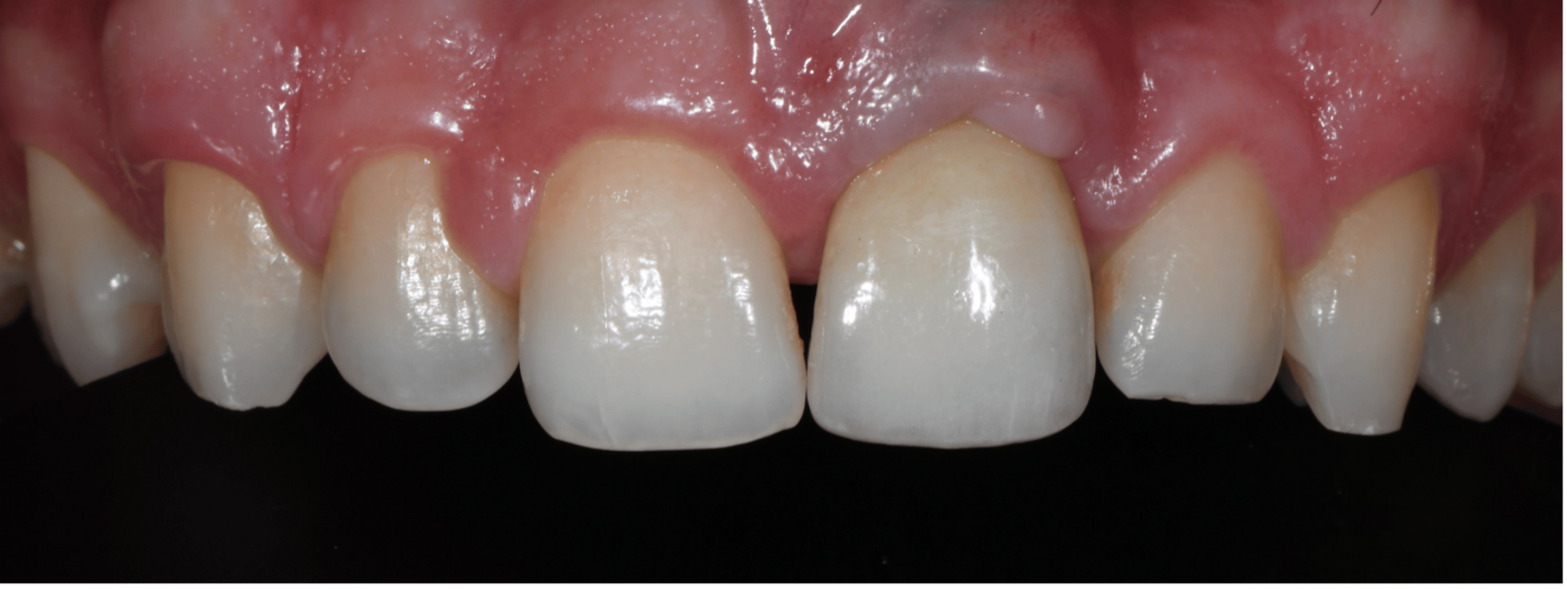
One-month follow-up. Inflammation-free soft tissue conditions are visible around the new implant suprastructure

The permanent customized abutment was seated and secured at 30 Ncm according to the manufacturer's recommendations. Since the screw access hole was located on the esthetic/facial aspect, a cement-retained crown was fabricated. After the screw access hole was sealed with Teflon tape and composite resin, the restoration was permanently cemented using resin-modified glass ionomer cement (Fujicem Evolve, GC, Tokyo, Japan), and occlusion was adjusted (Figure 10). To prevent cement extrusion, Teflon tape was precisely positioned between the abutment and the soft tissue during the procedure.

**Figure 11:**
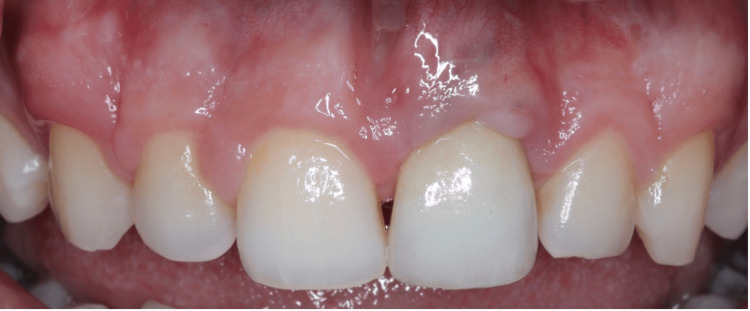
12-month follow-up. Note the ongoing peri-implant health and slight increase in papilla tissue between #11 and #21

**Figure 12:**
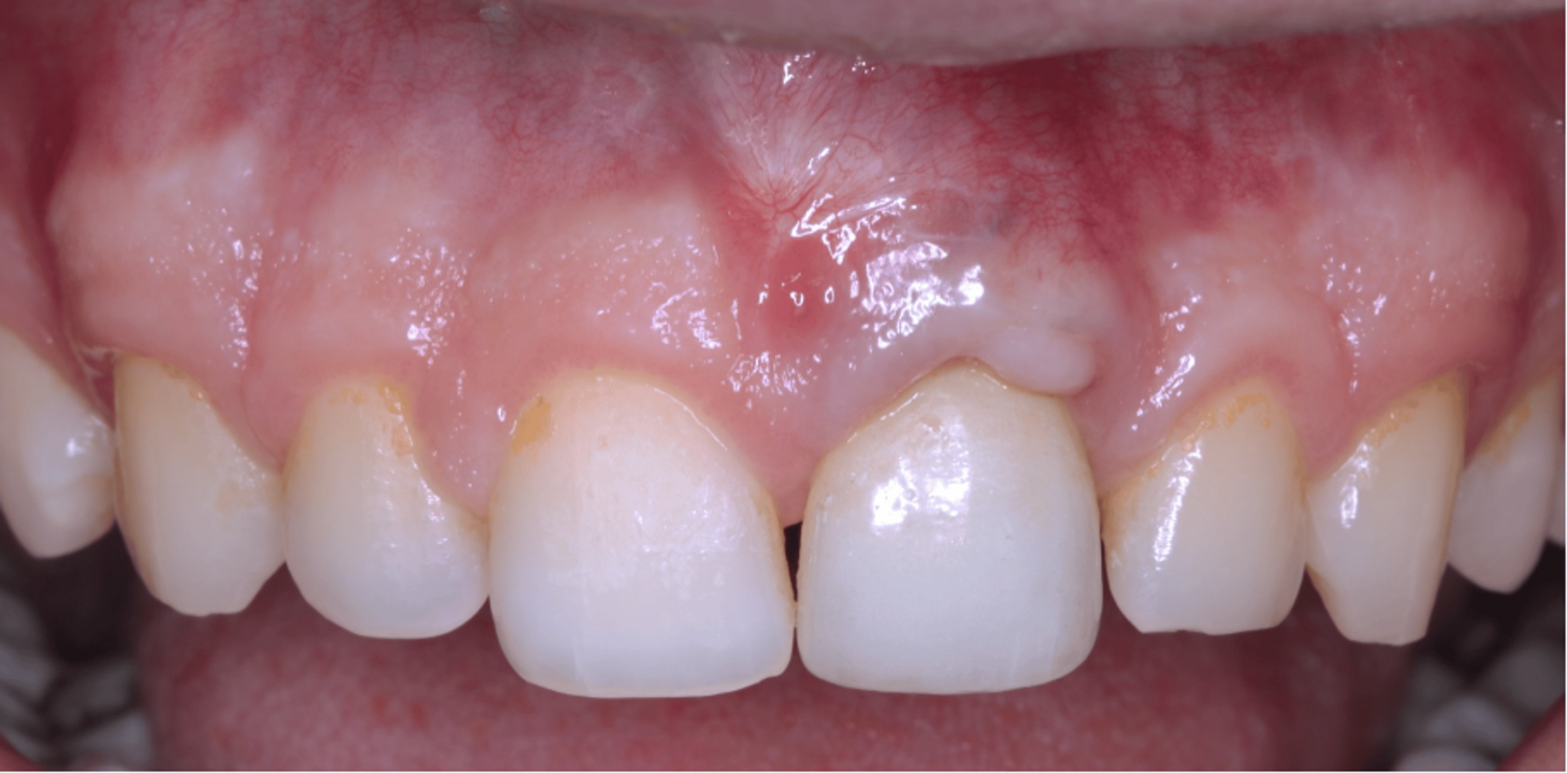
24-month follow-up. Matured gingival/peri-implant mucosa features (stippling, etc.) can be seen during the observational period

**Figure 13:**
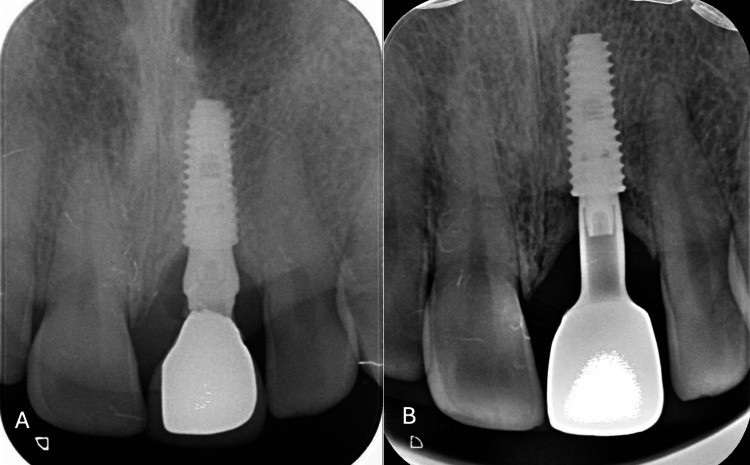
(A) Periapical X-ray at the beginning. (B) Periapical X-ray at the 30-month follow-up

The patient was followed up at one month, 12 months, 24 months, and 30 months following final crown delivery and received prophylactic treatment during the annual visits. At the one-month follow-up, inflammation-free soft tissue conditions were observed around the implant suprastructure (Figure 10). At the 12-month follow-up, ongoing peri-implant health was noted with a slight increase in papilla tissue between teeth #11 and #21 (Figure 11). At the 24-month follow-up, matured gingival and peri-implant mucosal features were observed (Figure 12). At the 12-month and 24-month follow-up visits, PD of 5 mm were recorded at the disto-buccal and disto-palatal aspects with no BOP, and the remaining sites maintained PD of 3 mm without bleeding. At the 30-month follow-up, these favorable peri-implant parameters were maintained. PD decreased from 8 mm at baseline to 5 mm at the disto-buccal and disto-palatal aspects, with no BOP at any site; the remaining sites maintained PD of 3 mm without bleeding. The Pink Esthetic Score (PES) was 11/14, and the White Esthetic Score (WES) was 8/10. The patient reported satisfaction with the esthetic and functional outcomes. Figure 13 shows increasing hard tissue support around the new custom abutment. A frenectomy was also performed at a subsequent follow-up visit following the stabilization of the soft tissue contour.

## Discussion

This case report presents a dental implant shortly after the traumatic loss of an anterior tooth during adolescence, which subsequently exhibited relative infraocclusion. Although immediate postoperative radiographs are not available, which constitutes a limitation of this report, the infraocclusion, along with other possible positional discrepancies, is presumed to have developed at least partially due to continued alveolar growth following implant placement during adolescence. However, an initial positioning error cannot be definitively excluded as a contributing factor. The concurrent presence of buccal malposition and infraposition is consistent with the growth-related changes reported in the literature for implants placed during the active growth period [2,9,24]. Three-dimensional (mesiodistal, buccolingual, and apicocoronal) implant positioning is of critical importance in achieving optimal esthetic and functional results, especially in the anterior region [25]. This case illustrates the potential consequences associated with deviations from the principles of three-dimensional implant positioning. In this case, the implant was positioned relatively deep and buccally within the alveolar ridge, which may contribute to compromised peri-implant health and long-term maintenance challenges. Deep implant placement may increase the complexity of restoration and may have implications for plaque control [25]. Buccal/facial positioning and axial inclination are common positioning errors in the bucco-oral dimension and have been associated with peri-implant mucosal recession [26]. However, these outcomes are multifactorial and should be interpreted with caution in the context of a single case.

In this case, a one-piece customized abutment was fabricated with a narrower diameter, thereby increasing the extent of platform switching. This design aimed to distance the inflammatory cell infiltrate, which has been reported as a source of inflammation at the implant-abutment connection in the subgingival zone, from the adjacent crestal bone [27-29]. Furthermore, the abutment maintained a uniform narrow diameter throughout the entire soft tissue corridor, expanding to the full crown width only in the final 2 mm of the subgingival area. The crown-to-implant ratio was maintained within acceptable limits to ensure biomechanical stability [27,30]. This configuration was intended to preserve the surrounding soft and hard tissues to the greatest extent possible. Indeed, comparison of periapical radiographs at baseline and the 30-month follow-up revealed an approximate coronal bone gain of 1.5-2 mm around the customized abutment, particularly in areas distant from the inflammatory zone. This finding supports the rationale for the platform-switching configuration employed in this case. Several systematic reviews and meta-analyses have demonstrated that platform switching significantly reduces marginal bone loss compared to platform-matched configurations, with the degree of bone preservation being proportional to the magnitude of the implant-abutment diameter mismatch [31,32]. The present case is of particular importance in that it demonstrates a positive clinical and radiographic effect of this approach, especially in situations where the implant is positioned more subgingivally than intended or, as in this case, has migrated to a subgingival position over time, and, moreover, that this effect was maintained over a medium-term follow-up period of 30 months.

The use of connective tissue grafts to compensate for mid-facial mucosal recession due to orofacial and/or axial malposition is a common consideration [25]. However, the predictability of connective tissue grafts usage in managing recession defects around implants is variable and depends on the degree of malposition [22,33,34]. The biologic properties of supportive graft tissue may affect its potential in this management. Harvesting a free gingival graft and de-epithelializing it (used as a connective tissue graft) is the most common way to obtain the dense connective tissue (lamina propria) which is the tissue of choice for connective tissue grafts [35]. In the present case, de-ep GG was used to support buccal soft tissue in order to improve the amount and stabilization of keratinized mucosa.

The buccally inclined position of the implant also resulted in insufficient soft tissue support around the abutment and a challenge in masking the gray reflection of the titanium customized abutment. Titanium abutments often have a grayish color that can compromise the esthetics of the restoration [36]. This abutment color can significantly affect the final shade of the restoration, particularly in cases with a thin mucosal phenotype. Options like anodizing the titanium and increasing the restoration's thickness or switching to a different abutment material have been suggested [37].

While customized zirconia abutments are preferred for their esthetic results, they have downsides, such as wear at the titanium-zirconia interface and a higher risk of fracture. Anodizing, on the other hand, provides a practical solution; a randomized controlled clinical trial reported no significant difference in patient-reported and clinician-assessed esthetic outcomes between anodized titanium and zirconia abutments in the anterior maxilla [38].

Anodization of titanium abutments has shown promising results, with studies indicating reduced bacterial adhesion compared to non-anodized surfaces while maintaining similar surface roughness [39]. It is also considered safe for gingival health, as in vitro studies suggest improved adhesion and proliferation of epithelial and gingival fibroblast cells [40]. Although clinical evidence is still limited, these findings support the potential of anodized abutments for enhanced long-term performance, especially in esthetically sensitive areas [41]. It should be noted that the sequential dual-voltage anodization protocol employed in this case, applying 80V to achieve a pink hue in the subgingival zone and subsequently 50V to achieve a yellow hue in the supragingival zone, is consistent with the dual-zone color anodization concept described in the literature, representing a clinically adapted application of this technique to address the specific esthetic challenges encountered in this case [42].

Anterior restorations should replicate the shape and morphology of neighboring teeth for optimum results. CBCT scans were combined with the STL file of the plaster model in designing phase to replicate natural morphology. This enabled the design of a customized abutment-crown integrated provisional restoration that matched the emergence profile of the contralateral tooth, which may be useful in esthetically demanding cases. In the present case, this approach contributed to a favorable emergence profile match, as reflected by a PES of 11/14 and a WES of 8/10 at the 30-month follow-up, along with patient-reported satisfaction with the esthetic outcome [43]. Also, traditional shade matching methods, such as using dental shade guides and intra-oral photographs, frequently lead to inconsistent and subjective results. This is influenced by factors like tooth dehydration during procedures, variations in ambient lighting, the operator's level of experience, and individual differences in visual perception. To address these challenges, instrumental methods like spectrophotometers and colorimeters have been introduced. While these tools are more expensive and require additional training, they offer a more objective and accurate approach to shade matching, minimizing errors and improving outcomes compared to conventional methods [43].

This case report has several limitations that should be acknowledged. First, the absence of immediate postoperative radiographs following the initial implant placement at age 15 prevents definitive differentiation between growth-related infraposition and initial malposition. Second, the follow-up period of 30 months, while sufficient to observe mid-term outcomes, does not allow for conclusions regarding the long-term stability of the soft tissue augmentation, abutment anodization, and prosthetic rehabilitation. Third, this is a single-case report, and the findings cannot be generalized; well-designed long-term prospective studies are needed to validate the efficacy of these combined strategies. Fourth, PES and WES could not be assessed at baseline due to the absence of a crown, limiting the ability to quantify esthetic improvement over time. Fifth, clinical photographs were taken under variable conditions across different timepoints, which limits the standardized visual comparison of soft tissue outcomes between baseline and follow-up. Finally, the unavailability of prefabricated prosthetic components with ideal gingival heights for such deeply positioned implants represents a practical limitation that necessitated clinical modifications, including the use of an open-tray impression coping as a gingiva former substitute and a longer scan body; such adaptations, while effective in this case, may not be universally applicable and highlight the need for expanded prosthetic component availability for complex implant positions.

## Conclusions

This case report illustrates the challenges of implant placement during adolescence, including progressive infraocclusion and compromised peri-implant soft tissue esthetics. The treatment approach involved soft tissue augmentation and anodization of a customized titanium abutment with an enhanced platform-switching concept, yielding favorable clinical and radiographic outcomes, including stable marginal bone levels, over a 30-month follow-up. However, these findings should be interpreted in light of the limitations inherent to case-based evidence. This case also highlights that implant rehabilitation in adolescents with tooth loss is generally recommended to be postponed until the completion of skeletal growth and the establishment of optimal clinical conditions. Nevertheless, the timing of implant placement should be individualized based on comprehensive growth assessment, as premature placement may result in significant positional discrepancies, compromising long-term peri-implant health, soft tissue stability, and esthetic outcomes.
